# Vaulting further: cranial vault expansion for craniocerebral disproportion without primary craniosynostosis

**DOI:** 10.1007/s00381-024-06517-2

**Published:** 2024-06-26

**Authors:** Jinggang J. Ng, Linda M. Saikali, Zachary D. Zapatero, Benjamin B. Massenburg, Meagan Wu, Dominic J. Romeo, Gregory G. Heuer, Scott P. Bartlett, Jesse A. Taylor, Jordan W. Swanson, Shih-Shan Lang

**Affiliations:** 1 Division of Plastic, Reconstructive and Oral Surgery, Children’s Hospital of Philadelphia, Philadelphia, PA USA; 2 Division of Plastic Surgery, University of Pennsylvania Perelman School of Medicine, Philadelphia, PA USA; 3 Division of Neurosurgery, Children’s Hospital of Philadelphia, Department of Neurosurgery, University of Pennsylvania Perelman School of Medicine, 3401 Civic Center Blvd, Philadelphia, PA 19104 USA

**Keywords:** Cranial vault expansion, Shunted hydrocephalus, PVDO, Idiopathic intracranial hypertension, Craniocerebral disproportion

## Abstract

**Purpose:**

Treatment of subjects with refractory idiopathic intracranial hypertension (IIH) or shunted hydrocephalus with chronic shunt complications is challenging. What is the role for cranial vault expansion, particularly utilizing posterior vault distraction osteogenesis (PVDO), in these cases? This study assesses medium-term efficacy of cranial vault expansion in this unique patient population.

**Methods:**

A retrospective review was conducted of patients who underwent cranial vault expansion from 2008 to 2023 at the Children’s Hospital of Philadelphia. Subjects who did not have a diagnosis of primary craniosynostosis were included in the study. Demographic information, medical history, and perioperative details were collected from medical records. Primary outcomes were the rate of CSF diversion procedures and resolution of presenting signs and symptoms. Secondary outcomes were perioperative and 90-day complications and reoperation requirement.

**Results:**

Among 13 included subjects, nine (69.2%) patients had a primary diagnosis of shunted hydrocephalus and 4 (30.8%) patients had IIH. Twelve (92.3%) subjects underwent posterior vault distraction osteogenesis (PVDO) and one (7.7%) underwent posterior vault remodeling (PVR). All 4 patients with IIH demonstrated symptomatic improvement following PVDO, including resolution of headaches, vomiting, and/or papilledema. Among 9 patients with shunted hydrocephalus, CSF diversion requirement decreased from 2.7 ± 1.6 procedures per year preoperatively to 1.2 ± 1.8 per year following cranial vault expansion (*p* = 0.030). The mean postoperative follow-up was 4.1 ± 2.1 years and four (30.8%) patients experienced complications within 90 days of surgery, including infection (*n* = 2), CSF leak (*n* = 1), and elevated ICP requiring lumbar puncture (*n* = 1). Four (30.8%) patients underwent repeat cranial vault expansion for recurrence of ICP-related symptoms. At most recent follow-up, 7 of 9 patients with shunted hydrocephalus demonstrated symptomatic improvement.

**Conclusion:**

Cranial vault expansion reduced intracranial hypertension-related symptomology as well as the rate of CSF diversion-related procedures in patients with refractory IIH and shunted hydrocephalus without craniosynostosis, and should be considered in those who have significant shunt morbidity.

## Introduction

Cranial vault expansion is the clinical standard for the treatment of craniosynostosis, and is well documented to relieve elevated intracranial pressure (ICP) and prevent the associated downstream sequelae of untreated intracranial hypertension [1, 2]. However, other causes of craniocerebral disproportion such as idiopathic intracranial hypertension [3] (IIH) and slit ventricle syndrome [4] (SVS) or shunted hydrocephalus requiring multiple and increasing shunt revisions do not have consensus treatment strategies. Treatment for IIH often begins with medical management through weight loss for overweight patients and/or drug therapy [5]. In refractory cases, surgical management may be necessary via cerebrospinal (CSF) diversion [5], though these procedures may be associated with significant perioperative and long-term morbidity [5–8]. Similarly, in the treatment of slit ventricle syndrome—which is believed to result from CSF overdrainage from shunt placement [9]—or a patient with recurrent shunt revisions, a standardized therapeutic algorithm has yet to be established, and existing approaches include shunt revision, implant of an anti-siphon device, shunt pressure adjustment, subtemporal decompression, lumboperitoneal (LP) shunting, and/or cranial vault expansion [10–17]. However, these options may be accompanied by significant surgical morbidity without satisfactory resolution of presenting symptoms. [10–17]

Several studies have reported on the use of cranial vault expansion in the management of craniocerebral disproportion without primary craniosynostosis [16, 18–20]. Recent evidence from our institution suggested a comparable perioperative safety profile of posterior vault distraction osteogenesis (PVDO) between patients with craniocerebral disproportion without a primary synostotic condition (i.e., IIH or SVS) and those with syndromic craniosynostosis [16]. In that study, the authors also reported a significant decrease in shunt operations following PVDO at a median short-term follow-up of 3 years. [16] However, the literature on cranial vault expansion in IIH and SVS remains scarce, and little is known about the long-term efficacy of this treatment modality in providing symptomatic relief and reducing CSF diversion-related morbidity.

This study aims to report on the medium-term efficacy of cranial vault expansion in the treatment of subjects with IIH or SVS without primary craniosynostosis. Our hypothesis was that this intervention would resolve presenting symptoms and reduce CSF diversion-related morbidity. Here, we describe the largest series to-date of cranial vault expansion for craniocerebral disproportion without primary major suture craniosynostosis.

## Methods

This study was performed following approval from the Institutional Review Board at the Children’s Hospital of Philadelphia and in accordance with the principles of the Declaration of Helsinki. A retrospective review was conducted of all patients who underwent cranial vault expansion from 2008 to 2023. Subjects who did not have a diagnosis of major suture craniosynostosis, as confirmed by computed tomography (CT) scan, were included in the study. Demographic information, medical history, and perioperative details were collected from medical records. Primary outcomes were change in rate of CSF diversion procedures and resolution of symptoms related to increased intracranial hypertension, including headaches, visual disturbance, papilledema, and vomiting. Secondary outcomes were perioperative and 90-day complications and reoperation requirement. Descriptive analyses were performed on demographic data, and a one-tailed paired t-test was used to compare continuous variables. All statistical analyses were conducted using JASP (Version 0.18.1, JASP Team, 2023) with significance defined as *P* < 0.05.

## Results

Of 13 included subjects, 8 (61.5%) were male and 10 (76.9%) were White (Table 1). Nine patients (69.2%) had a primary diagnosis of shunted hydrocephalus and 4 (30.8%) patients had IIH. Two patients with IIH had a concomitant diagnosis of minor squamosal suture synostosis without scaphocephaly. Ten (76.9%) patients had a ventriculoperitoneal (VP) shunt at the time of initial vault expansion.

**Table 1:** Cohort characteristics^a^

Patient characteristics (*N* = 13)	*N* (%)
Sex	
Male	8 (61.5)
Female	5 (38.5)
Race	
White	10 (76.9)
Black or African American	1 (7.7)
Native Hawaiian or Pacific Islander	1 (7.7)
Other	1 (7.7)
Ethnicity	
Non-Hispanic or Latino	12 (92.3)
Hispanic or Latino	1 (7.7)
Indication for cranial vault expansion	
Slit ventricle syndrome	9 (69.2)
Idiopathic intracranial hypertension	4 (30.8)
Age at initial surgery (years)	5.5 ± 3.6
Length of hospitalization (days)	9.7 ± 6.5
Postoperative follow-up (years)	4.1 ± 2.1

^a^Continuous data presented as mean ± standard deviation

Twelve (92.3%) subjects underwent PVDO and one (7.7%) underwent posterior vault remodeling (PVR). Among 11 patients who underwent PVDO, 8 underwent traditional PVDO with anteroposterior expansion and 3 underwent transverse/multivector PVDO. Mean age at initial surgery was 5.5 ± 3.6 years, and mean length of hospitalization was 9.7 ± 6.5 days. There were no apparent intraoperative complications among the cohort. Mean postoperative follow-up was 4.1 ± 2.1 years. Four (30.8%) patients experienced complications within 90 days of surgery, including infection in 2 patients that resolved with antibiotic therapy, and CSF leak in one patient that was likely related to external ventricular drain (EVD) removal in the same time period. Additionally, one patient experienced worsening headaches, blurry vision, and emesis with demonstrated papilledema and elevated opening pressure on lumbar puncture in the postoperative period, with resolution of symptoms over time. Among subjects who underwent PVDO, mean-elapsed time between distractor placement and removal was 3.1 ± 0.6 months.

### IIH cohort

**Fig. 1:**
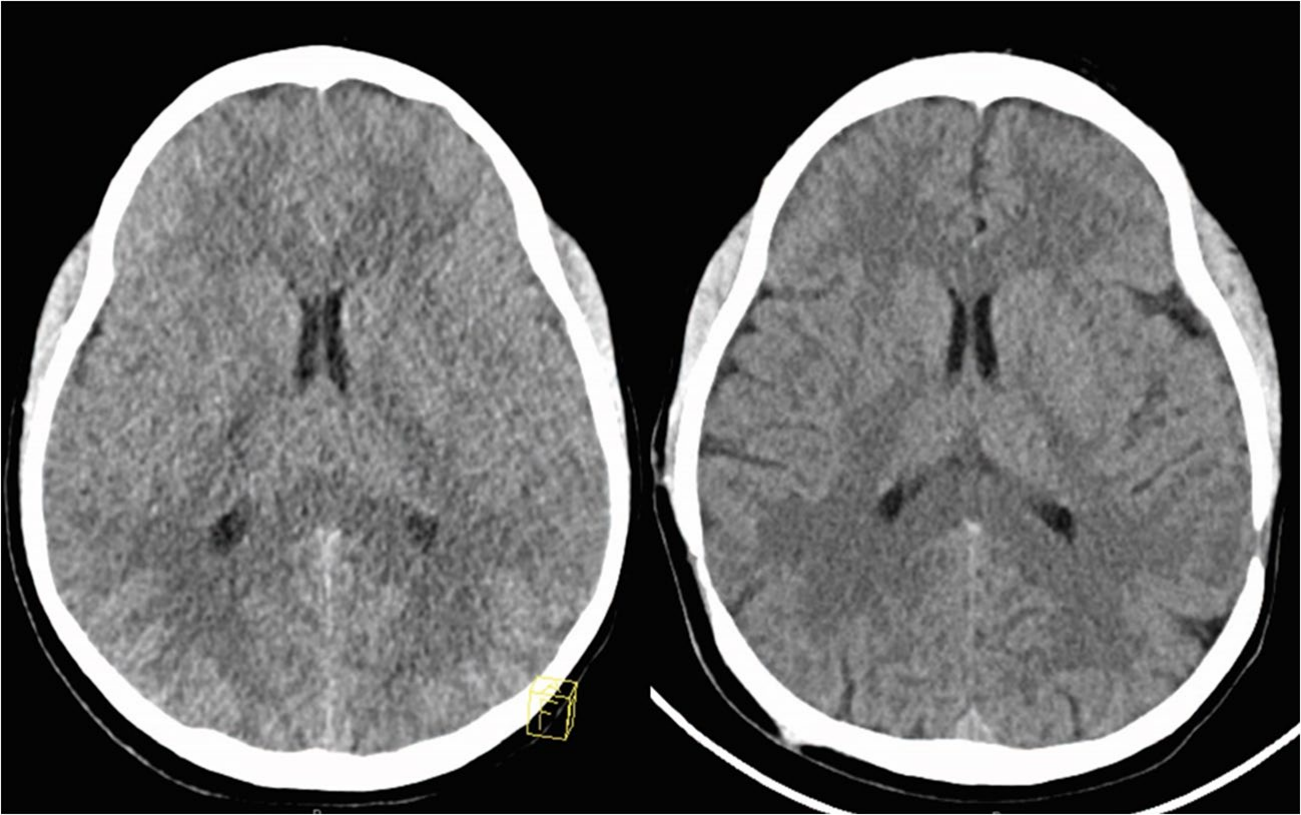
Computed tomography (CT) scan of a patient with idiopathic intracranial hypertension (IIH) **a** prior to and **b** following posterior vault distraction osteogenesis (PVDO)

**Fig. 2:**
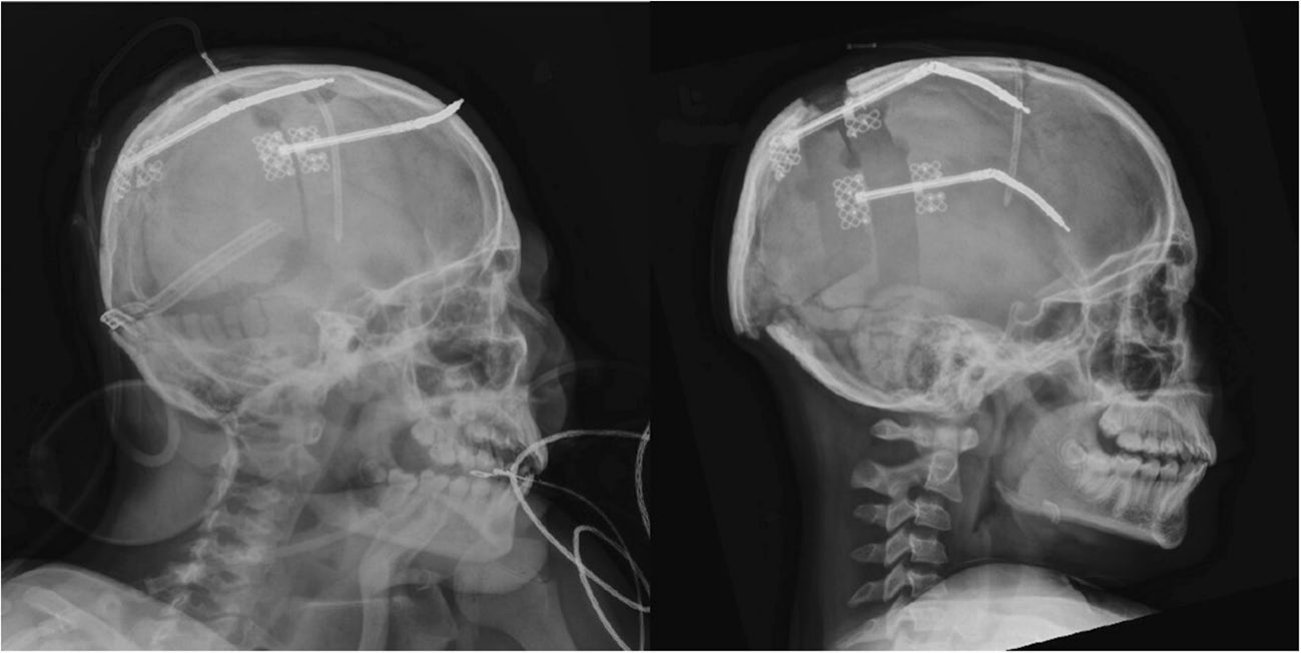
Lateral cephalogram of a patient with idiopathic intracranial hypertension (IIH) **a** following distractor placement and **b** prior to distractor removal for posterior vault distraction osteogenesis (PVDO)

Among 4 patients with IIH, mean body mass index (BMI) was 18.5 ± 4.7 at the time of PVDO. Three underwent cranial vault expansion after failing medical management of ICP-related symptoms (Figs. 1, 2). Following discussion with patients and their families, who expressed desire to avoid CSF diversion-related morbidity, PVDO was offered as a potential treatment approach. All 4 patients with IIH demonstrated symptomatic improvement, including resolution of headaches, vomiting, and/or papilledema. None of these patients underwent repeat cranial vault expansion at last follow-up. One of the 4 patients had experienced several VP shunt-related infections within a few weeks of surgery, and since he was not yet shunt-dependent, PVDO was proposed as an alternative. As of his most recent follow-up at 1.6 years postoperatively, this patient did not require a VP shunt.

### Shunted hydrocephalus cohort

**Fig. 3:**
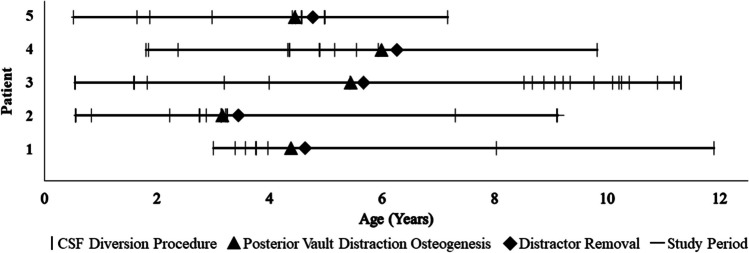
Figure depicting cerebrospinal fluid (CSF) diversion (vertical line), posterior vault distraction osteogenesis (triangle), and distractor removal (diamond) of patients with shunted hydrocephalus who underwent a single cranial vault expansion in the study period

Among 9 patients with shunted hydrocephalus, hydrocephalus etiologies were: tumor, 2 (22.2%); trauma, 1 (11.1%); post-hemorrhagic hydrocephalus, 1 (11.1%); congenital, 1 (11.1%); post-surgery, 1 (11.1%); vein of Galen, 1 (11.1%); arachnoid cyst and post-surgery, 1 (11.1%); and pseudotumor, 1 (11.1%). Eight patients underwent PVDO and one underwent PVR due to parental concern regarding external hardware and the daily distraction requirement. CSF diversion requirement decreased from 2.7 ± 1.6 procedures per year preoperatively to 1.2 ± 1.8 per year following initial cranial vault expansion (*p* = 0.029) (Fig. 3). Types of shunt devices used included Medtronic Strata adjustable pressure valve (Medtronic, Dublin, Ireland) (*n* = 9 patients), Medtronic Delta valve (Medtronic, Dublin, Ireland) (*n* = 5), MIETHKE proGAV position-dependent valve (Christoph Miethke GmbH, Potsdam, Germany) (*n* = 4), Medtronic fixed-pressure valve (Medtronic, Dublin, Ireland) (*n* = 1), and Codman Certas Plus programmable valve (Integra Life Sciences, Princeton, New Jersey, USA) (*n* = 1). Four (30.8%) patients underwent repeat cranial vault expansion for recurrence of presumed ICP-related symptoms, which included headaches, papilledema, and/or change in mental status. Among those requiring reoperation, 3 underwent fronto-orbital advancement due to the presence of anterior cranial dysmorphology, and one underwent repeat PVDO; in 3 of these patients, symptoms resolved following repeat cranial vault expansion. At most recent follow-up, all 9 subjects had a VP shunt in place, and 7 demonstrated symptomatic improvement of their ICP-related symptoms.

## Discussion

Several important findings emerged from this study of the effectiveness of cranial vault expansion in alleviating CSF diversion-related morbidity and presenting symptomatology in children with IIH or SVS. Among subjects with IIH, PVDO was associated with long-term symptomatic relief. Although a few patients underwent repeat cranial vault expansion, patients with shunted hydrocephalus still demonstrated a significant reduction in the rate of CSF diversion following PVDO/PVR.

Prior reports have investigated the use of cranial vault expansion in the treatment of craniocerebral disproportion without primary craniosynostosis [16, 18–20]. However, there is limited evidence on the long-term efficacy of this modality in treating IIH and SVS and/or shunted hydrocephalus. In the management of IIH, the goal of therapy is reduction of elevated ICP to preserve vision and resolve associated symptoms [3]. First-line treatment for this condition is weight loss in overweight patients [21, 22] and/or drug therapy with acetazolamide [3, 5, 23]. However, the efficacy of these treatments is limited; weight loss may be difficult and/or unsuitable particularly in the pediatric population in whom IIH is often unrelated to weight [5, 24–26]. Furthermore, certain medications can be associated with significant side effects [23, 27]. These challenges are compounded in young children, for whom weight loss and/or long-term drug therapy may be inappropriate or intolerable.

In cases of IIH refractory to medical management, surgical intervention may be pursued via optic nerve sheath fenestration (ONSF)—a procedure in which the optic nerve sheath is incised to enable CSF drainage and reduce pressure on the optic nerve—and/or CSF diversion through LP or VP shunting [5]. While most children experience symptomatic improvement following CSF diversion [28, 29], these procedures can be associated with significant complications and long-term morbidity including infection, obstruction, tonsillar herniation, and lumbar radiculopathy depending on the location of the shunt [5–8, 30]. Moreover, children may be particularly susceptible to shunt-related complications, possibly due to shunt migration from natural growth or the relative size of shunt tubing in the thecal sac in an LP shunt [6]. In some patients with IIH, symptoms are left unresolved by conventional medical therapy, ONSF, and/or CSF diversion. [5]

In those who failed medical management and desire to avoid/reduce morbidity related to CSF diversion, cranial vault expansion may be considered as a treatment modality. In our cohort, 4 patients underwent PVDO for IIH after failing conventional management. Among these patients, all demonstrated symptomatic improvement at long-term follow-up. Moreover, one patient who had previously experienced numerous shunt-related infections was shunt-free at most recent follow-up. A previous study reported symptomatic improvement in 2 of 3 patients who underwent PVDO for IIH at a median follow-up of 3 years. [16] Our findings add to existing literature by providing a longer-term view on the efficacy of PVDO in the treatment of IIH.

Similarly, slit ventricle syndrome—a chronic complication of CSF diversion—and shunted hydrocephalus with multiple recurrent shunt revisions are challenging conditions for which there is no consensus treatment strategy [4]. Existing literature describes potential treatment approaches ranging from shunt revision, addition of an anti-siphon device, shunt pressure adjustment, subtemporal decompression, LP shunting, to cranial vault expansion [10–17]. However, these procedures do not reliably resolve symptoms and may still require many reoperations [10–17]. Furthermore, much of existing literature on the management of SVS consists of case reports or series with limited or varied follow-up; thus, the efficacy of existing treatment modalities is not well understood. [4]

Cranial vault expansion for SVS/shunted hydrocephalus is not a novel treatment approach. An early report from our center studied 12 patients with “slit ventricle syndrome” and post-shunt craniosynostosis and demonstrated improvement in neurologic symptoms following cranial vault expansion via bilateral fronto-orbital advancement with frontotemporoparietal expansion or PVR [31]. However, 10 of these patients had radiologically-confirmed craniosynostosis of major calvarial sutures, while 2 had “functional” synostoses with narrowed/overlapping sutures [31]. More recently, our center published a report discussing the short-term outcomes of 6 patients who underwent PVDO for slit ventricle syndrome [16]. In contrast to the earlier study, none of these 6 patients had a primary diagnosis of craniosynostosis [16]. Additionally, since the publication of the early report, our center adopted PVDO as a preferred modality for cranial vault expansion in patients with cephalocranial disproportion without primary craniosynostosis. In the more recent study, patients with SVS demonstrated a significant reduction in shunt rate at a median follow-up of 3 years. [16] Our findings suggest that this reduction in CSF diversion procedures following cranial vault expansion is sustained at medium-term follow-up.

Notably, almost half of the SVS/shunted hydrocephalus subgroup underwent repeat cranial vault expansion, suggesting that in a portion of these patients, initial PVDO may not be a definitive solution. The presence of anterior dysmorphology in several of these patients suggests that appearance-related concerns may have also played a role in decision-making for additional surgery. However, this sample may be biased as these patients continued to have symptomatology often without clear signs of elevated ICP, and therefore were difficult to manage. Nonetheless, 7 of 9 patients in our cohort reported symptomatic improvement at most recent follow-up.

**Fig. 4:**
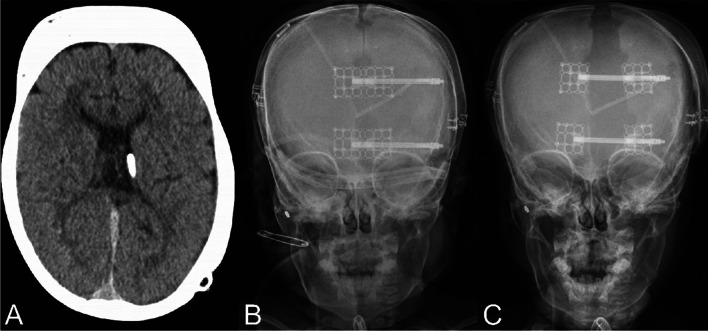
Patient with shunted hydrocephalus **a** preoperatively (computed tomography (CT) scan), **b** following distractor placement (frontal cephalogram), and **c** prior to distractor removal (frontal cephalogram) for posterior vault distraction osteogenesis (PVDO) with transverse distraction

**Fig. 5:**
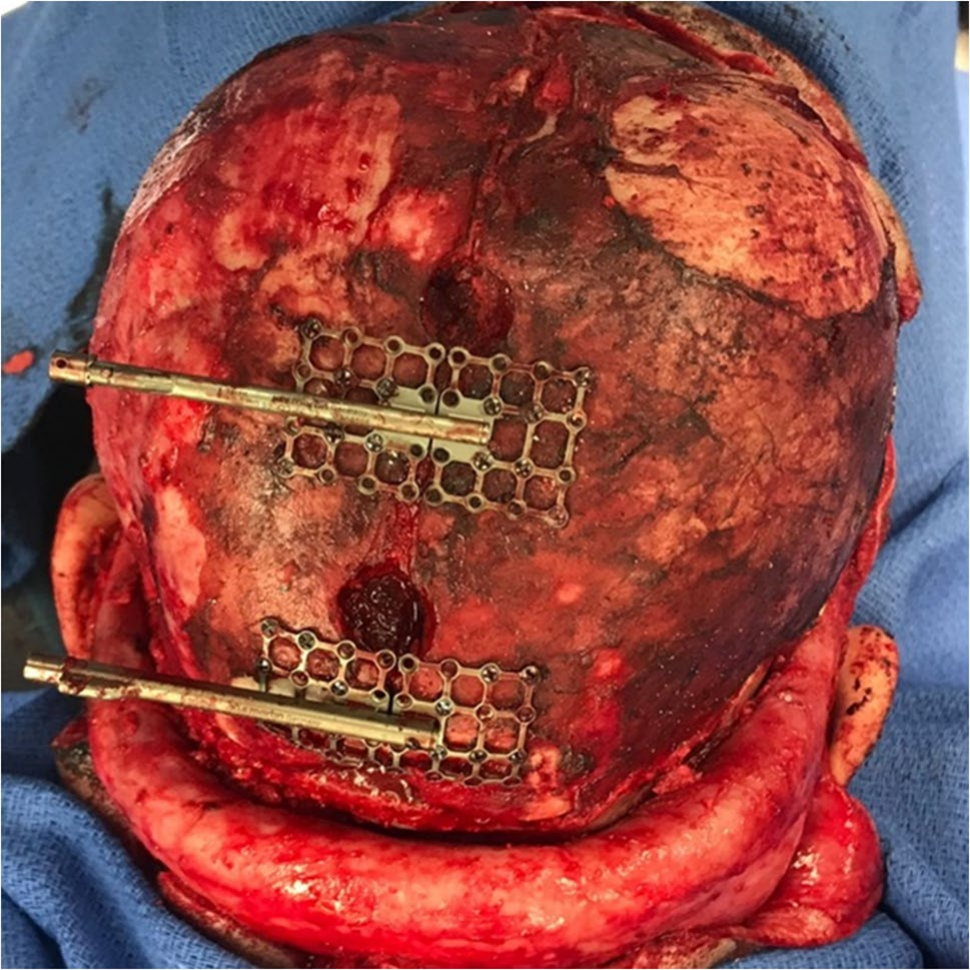
Intraoperative photo illustrating distractor placement for subject depicted in Fig. 4

Importantly, in patients with normocephalic or baseline scaphocephaly, we have adopted transverse/multivector cranial vault distraction osteogenesis (CVDO) so as to improve (and not iatrogenically worsen) their cranial morphology (Figs. 4, 5). This modified surgical technique has previously been described by our unit [32] and features transverse expansion with or without anteroposterior lengthening. Because the expansion pattern from vertex view appears to show the spread of anteriorly-based parietal bone “wings” from the cranium, we like the term “ladybug CVDO,” first suggested by Dr. Jo Barta. As reflected in our cohort, traditional PVDO with anteroposterior expansion [33] is most often used. But in select cases, transverse expansion is employed with posterior distractors, with or without standard longitudinal distractors. Although technically more complex and slightly less stable, we find that multivector (simultaneous anteroposterior and transverse expansion) PVDO yields the largest volume gains.

At our institution, PVDO is preferred to other forms of cranial vault expansion in patients with cephalocranial disproportion without primary craniosynostosis. There are several reasons underlying this choice. First, PVDO has demonstrated superior volumetric gains in comparison to anterior vault expansion [34, 35]. Second, distraction osteogenesis may increase the degree of expansion that can be achieved via gradual stretching of soft tissue, minimize cranial defects through gradual ossification between bone segments, and reduce complications associated with dead space created by large movements [36–39]. Finally, preserving the virgin frontal bone allows for increased flexibility for future correction of frontal dysmorphology, as appropriate. Nonetheless, the risk of shunt malfunction and infection may be heightened in patients with VP shunts undergoing PVDO [40]. Additionally, in some patients with shunted hydrocephalus and/or chronic lung disease/respiratory pressure support, we observed increased thickness of bone and vascularity requiring additional hemostatic agents and bone wax intraoperatively [41]. Thus, the risk–benefit ratio of this intervention must be considered when determining treatment modality.

While cranial vault expansion is not considered first-line treatment for IIH or shunted hydrocephalus, in a select group of patients for whom other medical or surgical interventions have failed or caused significant morbidity, it may be a reasonable option within a surgeon’s armamentarium with promising medium-term outcomes. Potential limitations of this study include its small sample size and the subjective nature of neurological symptoms. Notwithstanding these constraints, we report on the largest series to-date of cranial vault expansion in craniocerebral disproportion without primary major suture craniosynostosis.

## Conclusions

Cranial vault expansion consistently reduces both intracranial hypertension-related symptomology and CSF diversion-related procedures in patients who have non-primary major suture craniosynostosis diagnoses of idiopathic intracranial hypertension or slit ventricle syndrome/shunted hydrocephalus. For a select group of patients, this treatment should be considered.
